# Switching charge-transfer characteristics from p-type to n-type through molecular “doping” (co-crystallization)†
†Electronic supplementary information (ESI) available: Additional schemes, figures and tables. Characterization of the complexes and pure DTPTP crystal. Details of the NMR, HR-MS, TGA, CV, TEM and calculations. CCDC 1439433 and 1439434. For ESI and crystallographic data in CIF or other electronic format see DOI: 10.1039/c5sc04954g


**DOI:** 10.1039/c5sc04954g

**Published:** 2016-02-25

**Authors:** Jing Zhang, Peiyang Gu, Guankui Long, Rakesh Ganguly, Yongxin Li, Naoki Aratani, Hiroko Yamada, Qichun Zhang

**Affiliations:** a School of Materials Science and Engineering , Nanyang Technological University , Singapore . Email: qczhang@ntu.edu.sg; b Division of Chemistry and Biological Chemistry , School of Physical and Mathematical Sciences , Nanyang Techno Logical University , Singapore; c Graduate School of Materials Science , Nara Institute of Science and Technology , Ikoma , Japan

## Abstract

Borrowing an idea from the silicon industry, where the charge-carrier's characteristics can be changed through heteroatom implantation, we believe that the charge transport nature of organic semiconductors can be switched through molecular “doping” (co-crystallization). Here, we report a novel molecule 2,7-di-*tert*-butyl-10,14-di(thiophen-2-yl)phenanthro[4,5-*abc*][1,2,5]thiadiazolo[3,4-*i*]phenazine (DTPTP), which originally is a p-type (0.3 cm^2^ V^–1^ s^–1^) compound, and can be switched to an n-type semiconductor (DTPTP_2_–TCNQ, 3 × 10^–3^ cm^2^ V^–1^ s^–1^ under air conditions) through tetracyanoquinodimethane (TCNQ) doping (co-crystallization). Single crystal X-ray studies revealed that TCNQ-doped DTPTP complexes (DTPTP_2_–TCNQ) adopt a dense one-dimensional (1D) mixed π–π stacking mode with a ratio of DTPTP and TCNQ of 2 : 1, while pure DTPTP molecules utilize a herringbone-packing pattern. Interestingly, theoretical analysis suggested that there is a quasi-2D electron transport network in this host–guest system. Our research results might provide a new strategy, to switch the charge transport characteristics of an original system by appropriate molecular “doping” (co-crystal engineering).

## Abstract 2

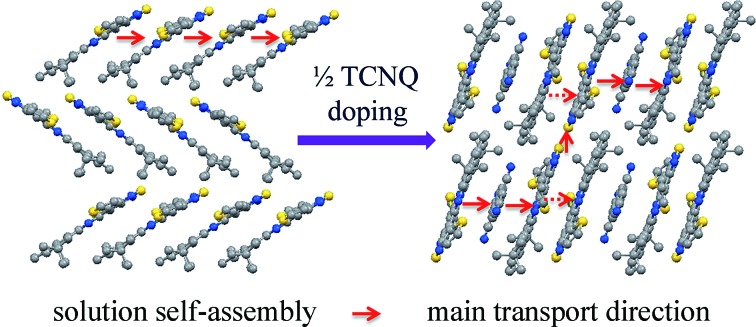
A novel molecule, DTPTP, which originally is a p-type compound, can be switched to an n-type semiconductor through tetracyanoquinodimethane doping (co-crystallization).

## Introduction

Organic semiconductors have attracted widespread interest due to their large range of applications including in radio frequency identification (RFID) tags,^1^ electronic papers,^2^ optical displays,^3^ various sensors,4a–d memory devices4e–f and organic photovoltaics.^5^ Compared with inorganic semiconductors, the structures and properties of organic conjugated materials as well as their solubility and packing during the operation process^6^ can be precisely manipulated through organic synthesis. However, a remaining bottleneck faced by organic synthetic chemists is the lack of accurate prediction of the concomitant properties, which has resulted in a lot of materials with undesired or even no performance. For example, although huge efforts have been put into preparing organic n-type semiconductors,^7^ few of them can meet the basic requirements (*e.g.* a suitable energy level and effective charge transport) for air-stable high performance devices.^8^ In addition, introducing too many electron-withdrawing elements (*e.g.* N atoms, –F, –CN and so on) into the backbone of known systems might dramatically change the electron density of the π-conjugated frameworks, which makes the targeted compounds extremely unstable. Thus, finding a new method to modify the known systems for property enhancement or charge-transport switching is highly desirable.^9^

Recently, the construction of multicomponent molecular solids based on molecular “doping” (crystal engineering) has offered a promising alternative way to alter the molecular arrangement, change the intermolecular interactions, and switch the charge transport characteristics, which are then completely different from the parent single-component materials.^10^ For example, 1 : 1 D–A co-crystals with either mixed stacking or segregated stacking have been prepared using a solution process and the as-prepared complexes displayed ambipolar transport properties,^11^ light emitting phenomena,^12^ photovoltaic effects^13^ and so on.^14^ The driving forces to form co-crystals during the self-assembly are believed to be π–π interactions, charge transfer, or H-bonds.^15^ Moreover, the remarkable and promising electronic properties of mixed stacking D–A charge-transfer (CT)-crystals have been theoretically predicted *via* quantum chemical calculations by Brédas and coworkers,^16^ who emphasized the significance of super-exchange along the stacking direction. Zhu *et al.* reported a 1 : 1 donor–acceptor co-crystal with meso-diphenyl tetrathia[22]annulene[2,1,2,1] (DPTTA) as the donor and extended π-conjugated 4,8-bis(dicyanomethylene)-4,8-dihydrobenzo[1,2-*b*:4,5-*b*′]-dithiophene (DTTCNQ) as the acceptor, which displayed remarkably high ambipolar transport with both the electron and hole mobility exceeding 0.1 cm^2^ V^–1^ s^–1^, attributed to the quasi-2D ambipolar transport network.^17^ However, other functional complexes with different ratios are rarely reported. One possible reason is that an imbalanced proportion of the prime components may disturb the inherent intermolecular interactions and induce ineffective transport channels. Up to now, the research on the careful selection of a host–guest pair with appropriate frontier molecular orbitals to achieve an efficient CT complex system is still in its infancy.

Here, we report the synthesis and characterization of a novel organic conjugated molecule 2,7-di-*tert*-butyl-10,14-di(thiophen-2-yl)phenanthro[4,5-*abc*][1,2,5]thiadiazolo[3,4-*i*]phenazine (DTPTP), which originally is a p-type compound (0.3 cm^2^ V^–1^ s^–1^), and can be switched to an n-type semiconductor (DTPTP_2_–TCNQ, mobility: 3 × 10^–3^ cm^2^ V^–1^ s^–1^ under air) through tetracyanoquinodimethane (TCNQ) doping (co-crystallization). Compared with traditional crystalline complexes, the advantage of this unprecedented sandwich supramolecular system is that both the hosts and guests can be assembled into single mixed columns, where the small guests are locked in by thiophene and butyl substituent groups. Such arrangements can strongly improve the molecule packing density, which will not only enhance the good stability of the supramolecular framework, but will also allow effective charge transport. So far, this is the first example with a different host–guest ratio and an optimized highly-ordered packing structure that displays effective charge transport.

## Results and discussion

**Fig. 1:**
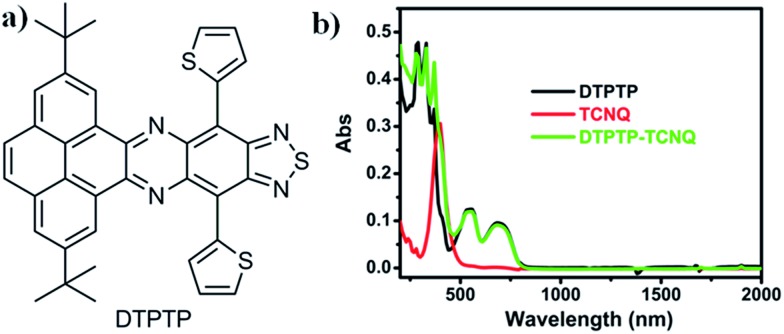
(a) Chemical structure of DTPTP; (b) UV-Vis-NIR spectra of the DTPTP–TCNQ mixture, DTPTP and TCNQ in toluene.

The synthetic route for the quasi-two dimensional D–A small molecule DTPTP (Fig. 1a) is provided in the ESI (Scheme S1†) and the as-prepared DTPTP has been fully characterized using NMR (Fig. S1†), HRMS (Fig. S2†), TGA (Fig. S3†), and single-crystal XRD. The single crystals of DTPTP_2_–TCNQ were obtained by slow evaporation of a toluene solution containing DTPTP and TCNQ (2 : 1 ratio).

The UV-Vis-NIR absorption spectrum of DTPTP in toluene was investigated (Fig. 1b). The optical energy bandgap of DTPTP was calculated to be –1.56 eV based on the onset of the absorption spectrum (796 nm), using the equation *E*_g_ = 1240/*λ*. The cyclic voltammetry measurements were performed with a three-electrode system (Fig. S4†). The LUMO energy level of DTPTP was estimated to be –3.91 eV, from the onset reduction potential with reference to Fc^+^/Fc (–4.8 eV) using the equation *E*_LUMO_ = –[4.8 – *E*_Fc_ + *E*onsetre] eV. The highest occupied molecular orbital (HOMO) energy level of DTPTP is –5.47 eV. These results suggest that DTPTP might have some possible applications in organic electronic devices. Interestingly, the UV-Vis-NIR spectrum (up to 2000 nm, Fig. 1b) of the mixed solution (toluene as the solvent) containing DTPTP and TCNQ (2 : 1) almost didn't show any new peaks or peak shifts on comparison with that of the individual DTPTP or TCNQ over a long wavelength range, which might suggest that no obvious charge-transfer process exists in the mixed solution.

**Fig. 2:**
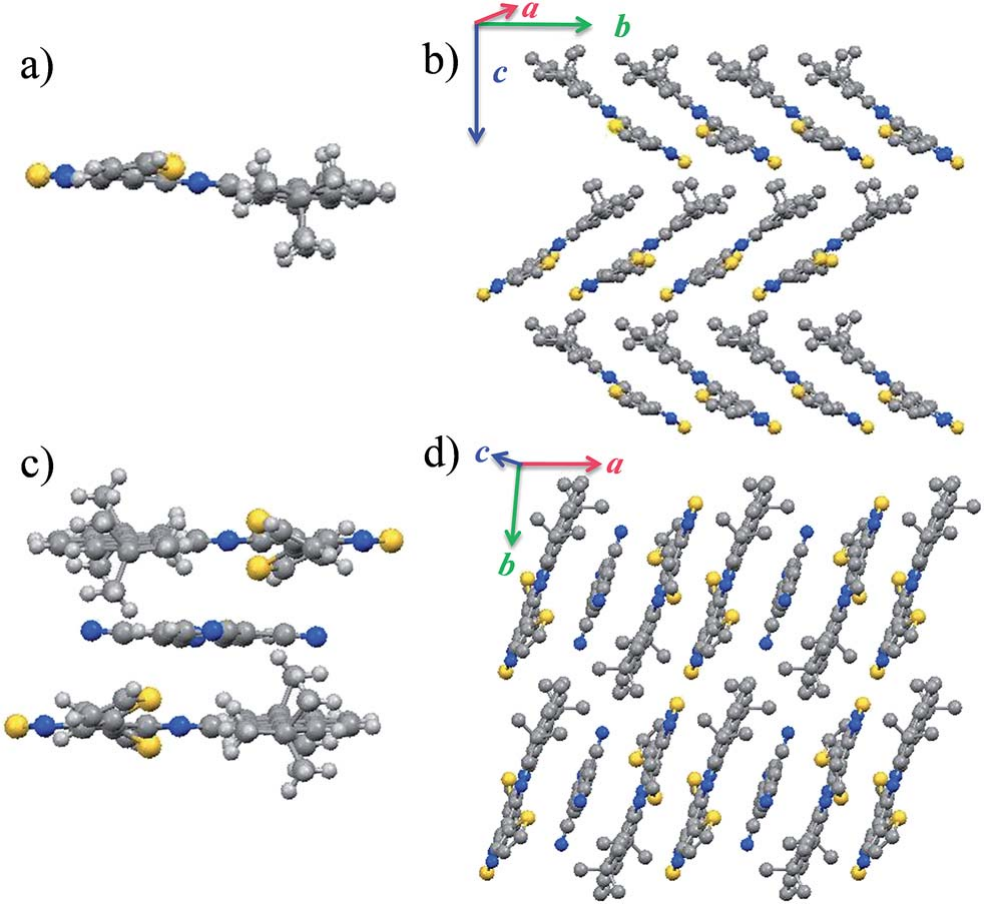
Crystal structures of DTPTP and DTPTP_2_–TCNQ: (a) molecular structure of DTPTP; (b) crystal packing of DTPTP (herringbone stacking pattern); (c) molecular structure of DTPTP_2_–TCNQ; (d) crystal packing of the host–guest system (π–π stacking pattern).

The crystal structure and the packing mode of DTPTP are shown in Fig. 2a and b (CCDC number: ; 1439433 †). Compound DTPTP crystallized in space group *P*2_1_/*c* of the orthorhombic system with unit-cell dimensions of *a* = 29.6046(8) Å, *b* = 11.8388(4) Å, *c* = 17.1781(4) Å, *α* = 90.00°, *β* = 90.00°, and *γ* = 90.00°. The center moiety of DTPTP adopted a slightly twisted structure with a dihedral angle of 5.8°, which was observed between the pyrene unit and the [1,2,5]thiadiazolo[3,4-i]quinoxaline (TQ) species. In this crystal structure, the conformations of the individual thiophene substituent groups deviate from the main backbone and both of them are bent towards the second molecule to tighten the stacking. Like the previously reported representative materials (pentacene and rubrene),^18^ the DTPTP molecules stack into a herringbone arrangement with π-stacking interactions along the *b*-axis direction, resulting in efficient electronic couplings and a transfer integral along this direction (Fig. 2b). DTPTP_2_–TCNQ crystallized in space group *P*1[combining macron] (triclinic system, CCDC number: ; 1439434 †) with unit-cell dimensions of *a* = 10.6919(2) Å, *b* = 11.6399(2) Å, *c* = 14.4791(3) Å, *α* = 90.178(6)°, *β* = 104.400(7)°, and *γ* = 95.311(7)°. Fig. 2c shows a component unit of the host–guest co-crystal, where two DTPTP molecules are wrapped above and below the TCNQ molecule, forming a cage to avoid the small additives escaping. The conformation of the main backbone slightly deviates from planarity with one thiophene from this molecule bending up and the other one bending down (the bending angle is about 26°). Besides, the large butyl groups on both sides also lock in the TCNQ molecules due to steric effects. It has been suggested that the degree of charge transfer in a complex can be estimated from the geometry of TCNQ.^19^ The ratio *c*/(*b* + *d*) relating the length of three bonds in TCNQ (indicated in Fig. S5†) was 0.478 for this complex, which is different from the value for neutral TCNQ (0.476). Here, *c* denotes the length of the isolated carbon–carbon double bonds in TCNQ; *b* and *d* correspond to the lengths of the carbon–carbon single bonds of the hexatomic ring and outer peripheral units, respectively. This result indicates that the charge transfer degree between DTPTP and TCNQ is about 0.1. The π–π stacking of the sandwich unit forms a linear column structure along the *a*-axis (Fig. 2d). Non-bonded interactions exist between the host and guest molecules along these columns. Host–host and host–guest short contacts were also found for neighboring columns (Fig. S6†).

Unlike other functional complexes (most with a 1 : 1 ratio) reported till now, the two DTPTP molecules and the TCNQ molecules stack alternatively into one-dimensional mixed columns along the stacking direction adopting a –H–H–G–H–H–G– mode. With the relatively small volume, every two adjacent DTPTP molecules are sandwiched with one TCNQ molecule. The head-to-tail stacking resulted in efficient overlap between the DTPTP planes, where the terminal pyrene group is considered to have less contribution to the π-conjugated TQ unit. Although there is no expected π-stacking among the guest molecules in the crystal, direct interaction effects were expected for the stacking columns, and lateral interactions for adjacent columns between the DTPTP molecules could also be observed, which could serve as the conducting channels.

**Fig. 3:**
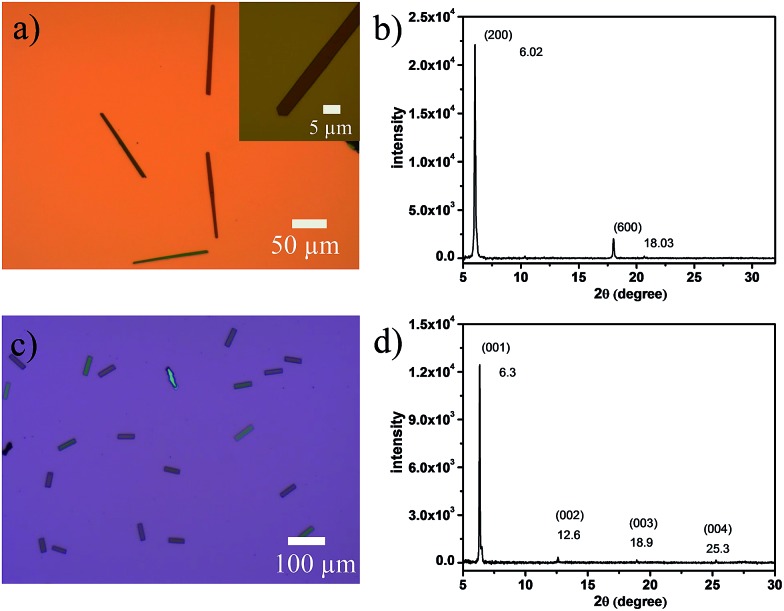
(a and c) Optical micrographs and (b and d) XRD patterns of the microcrystals of self-assembled (a and b) DTPTP and (c and d) DTPTP_2_–TCNQ. In (b) and (d), the peaks are indexed with lattice constants of the bulk crystals.

Single-crystalline microribbons/sheets were prepared by drop-casting their toluene solutions onto n-octadecyltrichlorosilane (OTS) modified SiO_2_ substrates. Optical microscopy (OM) observations revealed that the long DTPTP microribbons exhibit a regular hexagonal shape with tailored angles of 111° as shown in Fig. 3a. The lengths of the as-obtained crystalline microribbons are in the range of several tens to hundreds of micrometers. The corresponding X-ray diffraction pattern (Fig. 3b) shows intense peaks for (200) and (600) within the crystallographic data of DTPTP. In addition, transmission electron microscopy (TEM) characterization and corresponding selected area electron diffraction (SAED) (Fig. S7†) measurements were employed to elucidate the structure of the DTPTP microribbons. It could be concluded that all the single crystals grew along the (010) direction, which is the preferable charge transport direction (*b*-axis). Fig. 3c shows an OM image of DTPTP_2_–TCNQ parallelogram-like nanosheets, homogeneously covering the substrate with lengths of several tens of micrometers and thicknesses of tens to several hundred nanometers. For the DTPTP_2_–TCNQ nanosheets, the XRD pattern showed intense peaks at 6.3, 12.6, 18.9, and 25.3°, which were indexed as (001), (002), (003) and (004) within the crystallographic data of DTPTP_2_–TCNQ, suggesting that the crystals grow with the *ab* plane parallel to the substrate. The growth direction of the DTPTP_2_–TCNQ crystals was determined to be along the *a*-axis, the mixed stacking direction (Fig. S8†).

**Fig. 4:**
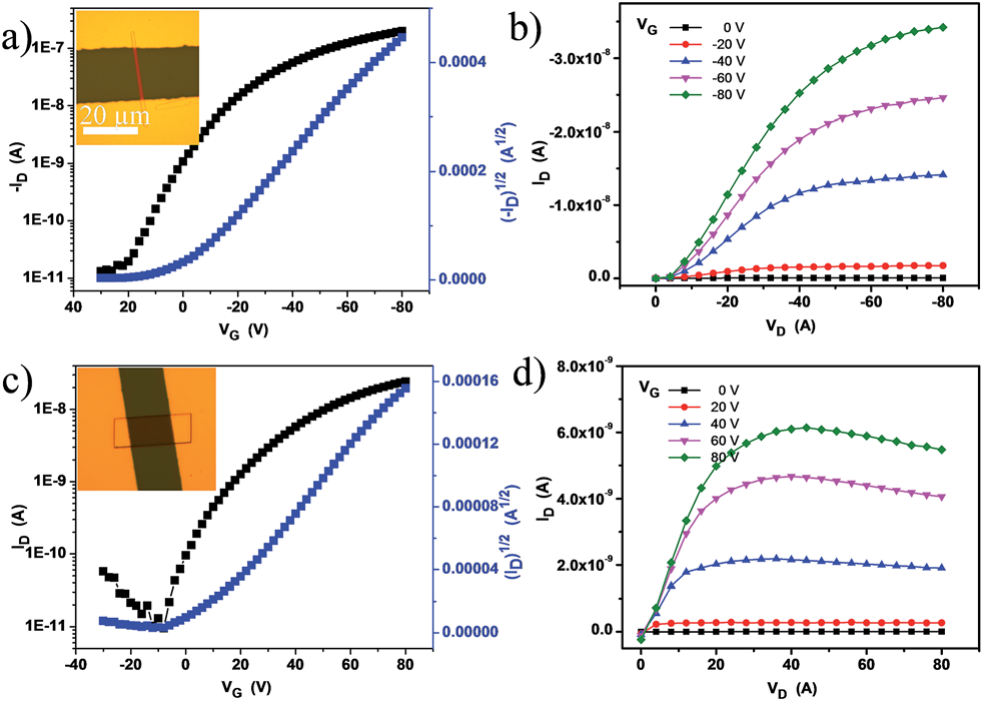
(a) Transfer and (b) output characteristics of the DTPTP single crystal ribbon device. (c) Transfer and (d) output characteristics of the device based on DTPTP_2_–TCNQ co-crystal nanosheets.

To investigate the influence of TCNQ insertion, we manufactured microcrystal transistor devices (for both DTPTP and DTPTP_2_–TCNQ) through thermally evaporating Au source/drain electrodes onto the microcrystals *via* a copper mask to meet the requirements of these single crystals. Copper grid masks were picked up using tweezers and placed over the prepared crystals. Subsequently, the source/drain electrodes were vacuum-deposited on the structure, and then the “copper grid masks” were peeled off and the crystal transistors were fabricated. The typically measured transfer and output characteristics of the devices are shown in Fig. 4. From the transfer characteristics, the devices based on the DTPTP single crystals exhibited a hole field-effect mobility of up to 0.3 cm^2^ V^–1^ s^–1^ and an on/off ratio of 10^5^ along the *b*-axis under atmospheric conditions (Fig. 4a and b). On the contrary, the devices based on DTPTP_2_–TCNQ nanosheets displayed typical electron transport properties, an electron mobility of around 0.003 cm^2^ V^–1^ s^–1^ and an on/off ratio above 10^4^ (Fig. 4c and d). No p-type behavior was observed during the characterization of the co-crystal devices and also no n-type performance was obtained for the DTPTP microcrystal devices. The performances of all the transistors were investigated under air, indicating the excellent stability under environmental conditions of the n-type complex transistors.

**Fig. 5:**
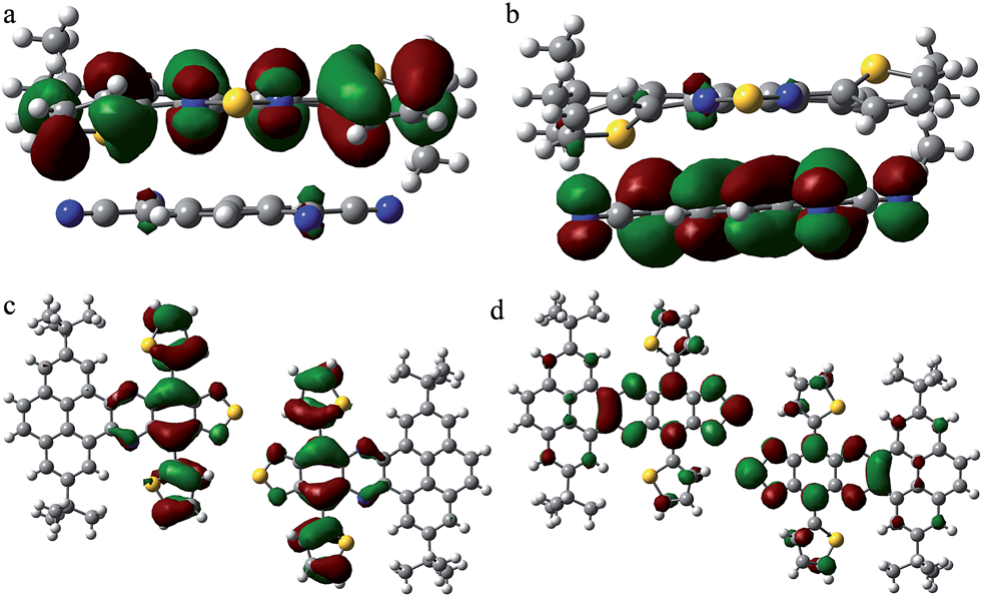
The (a) HOMO and (b) LUMO electron density distributions for a DTPTP:TCNQ dimer along the π–π stacking direction. The (c) HOMO and (d) LUMO electron density distributions for a DTPTP:DTPTP dimer along direction 11.

To understand the relationship between the molecular structure and charge transport properties for the unique DTPTP_2_–TCNQ host–guest system, the reorganization energies of DTPTP and TCNQ were calculated using a standard four-point method, and the intermolecular electronic couplings between neighbor molecules were obtained through a direct evaluation of the coupling element between the frontier orbitals using the unperturbed density matrix of the dimer Fock operator.^20^ The electron reorganization energies are 182.5 meV and 253.2 meV for DTPTP and TCNQ, respectively. The intermolecular electronic couplings, V (V_h_ for hole transfer and V_e_ for electron transfer), for all of the fourteen pathways (shown in Fig. S9†) were calculated at the DFT/PW91PW91/6-31G(d) level, and are given in Table S1.† The DTPTP:TCNQ dimer along the π–π stacking direction (direction 2, as shown in Fig. S9†) shows a large transfer integral of 19.58 meV for the electrons (Fig. 5a and b). To our surprise, the DTPTP:DTPTP dimer in adjacent columns along direction 11 (as shown in Fig. S9†) with five-membered rings approaching each other shows an even larger transfer integral of 25.76 meV for the electrons (Fig. 5c and d), which might be attributed to S–S intermolecular interactions, and thus construct a quasi-2D electron transport network. The main electron-transport pathways in the system contain the host–guest pair direction and the host–host pair direction, which indicates that the inserted TCNQ molecules are not only transporting electrons but also facilitating transport between DTPTP molecules due to the charge-transfer effect and the newly created packing arrangement. Overall, the transfer integrals for the electrons are larger than that of the holes, which indicates that DTPTP_2_–TCNQ facilitates electron transport other than hole transport. This is also consistent with our measured results.

## Conclusions

In conclusion, we have demonstrated that the charge transport characteristics of DTPTP can be switched from p-type to n-type through TCNQ “doping”. A 2 : 1 sandwich-like organic complex (DTPTP_2_–TCNQ) consisting of a small molecule TCNQ inserting into the center of two interlaced DTPTP planes has been obtained. Intermolecular charge-transfer interactions and the terminal groups (thiophene and butyl) cause the self-assembly of stable binary complexes into dense a one-dimensional mixed π–π stacking structure. Compared with pure DTPTP, which exhibited a p-type character (0.3 cm^2^ V^–1^ s^–1^), the FET devices based on DTPTP_2_–TCNQ single crystals exhibited n-type behavior with an electron mobility of up to 0.003 cm^2^ V^–1^ s^–1^ in an ambient atmosphere. Our results might provide new insights into the design and preparation of intelligent materials for high performance organic electronics and photoelectric devices.

## Experimental

### Materials

Tetrahydrofuran was dried with sodium before use and methylene chloride (DCM) was dried with calcium hydroxide before use. The other chemical reagents and solvents were purchased and used as received without further purification.

### Synthesis

Synthesis of 2,7-di-*tert*-butyl-10,14-di(thiophen-2-yl)phenanthro[4,5-*abc*][1,2,5]thiadiazolo[3,4-*i*]phenazine (DTPTP): a mixture of 4,7-di(thiophen-2-yl)benzo[*c*][1,2,5]thiadiazole-5,6-diamine (660 mg, 2 mmol) and 2,7-di-*tert*-butylpyrene-4,5-dione (688 mg, 2 mmol) in acetic acid (100 mL) and chloroform (20 mL) was stirred at 100 °C under nitrogen for 24 h. After cooling to room temperature, the mixture was poured into methanol and the crude product was purified using column chromatography over silica gel, eluting with dichloromethane/hexane to give purple crystals of the compound 2,7-di-*tert*-butyl-10,14-di(thiophen-2-yl)phenanthro[4,5-*abc*][1,2,5]thiadiazolo[3,4-*i*] phenazine (663 mg, 1.04 mmol, 52%).

^1^H NMR (300 MHz, CDCl_3_) *δ* 9.95 (d, *J* = 1.8 Hz, 2H), 9.15 (d, *J* = 3.6 Hz, 2H), 8.30 (d, *J* = 1.7 Hz, 2H), 8.02 (s, 2H), 7.84 (d, *J* = 5.2 Hz, 2H), 7.50–7.38 (m, 2H), 1.27 (s, 18H).

HR-MS, calcd for C_38_H_31_N_4_S_3_, 639.1711; found, 639.1694.

^13^C NMR could not be obtained due to the solubility of DTPTP.

### Growth of the micro-crystals and device fabrication

The SiO_2_/Si substrate was a heavily doped n-type Si wafer with a 500 nm thick SiO_2_ layer and a capacitance of 7.5 nF cm^–2^. The bare substrates were successively cleaned with pure water, piranha solution (H_2_SO_4_ : H_2_O_2_ = 2 : 1), pure water and then pure isopropanol. Treatment of the Si/SiO_2_ wafer with the OTS used in the present study was carried out using a vapor deposition method. The clean wafers were dried under vacuum at 90 °C for 0.5 h in order to eliminate the influence of moisture. After cooling to room temperature, a little drop of the OTS was placed on the wafers. Subsequently, this system was heated to 120 °C and the temperature maintained for 2 h under vacuum. Micrometer-sized single crystals of DTPTP_2_–TCNQ and DTPTP were obtained using a drop-casting method. After stirring DTPTP and TCNQ (2 : 1 ratio) in toluene for a long time (about two days), a mixed solution was obtained. A toluene solution containing DTPTP_2_–TCNQ or DTPTP (∼0.5 mg mL^–1^) was poured over the substrates and the solvent evaporated at room temperature. Drain and source Au electrodes (50 nm thickness) were deposited on the crystal by thermal evaporation with a copper grid as the shadow mask.

### Measurements

HR-MS (ESI) was recorded using a Waters Q-Tof premier™ mass spectrometer. ^1^H NMR spectra were recorded using a Bruker 300 MHz spectrometer. The investigation of the UV-vis absorbance was carried out with a Shimadzu UV-2501 spectrophotometer. Cyclic voltammetry was carried out with a CHI 604E Electrochemical Analyzer. Glassy carbon (diameter: 1.6 mm; area 0.02 cm^2^) was used as the working electrode, and platinum wires were used as the counter electrode and reference electrode, respectively. The potentials were recorded *versus* Pt in a solution of anhydrous DCM with 0.1 M tetrabutylammonium hexafluorophosphate (*n*-Bu_4_NPF_6_) as the supporting electrolyte at a scan rate of 100 mV s^–1^. Fc^+^/Fc was used as an internal standard, which has a HOMO energy level of –4.80 eV. Single-crystal diffraction analysis data were collected at 90 K with a BRUKER-APEX II X-ray diffractometer equipped with a large area CCD detector using graphite monochromated Mo-Kα radiation (*λ* = 0.71069 Å). The structures were solved and refined using a SHELXL-97 program. The hydrogen atoms were located at geometrically calculated positions and sometimes were not refined. The geometry structures were optimized using DFT calculations (B3LYP/6-31G*), and a frequency analysis was followed to ensure that the optimized structures were stable states. All calculations were carried out using Gaussian 09.^21^

### Quantum simulations

The electronic coupling for the holes and electrons can be obtained using Prof. Shuai's code based on eqn (1).1
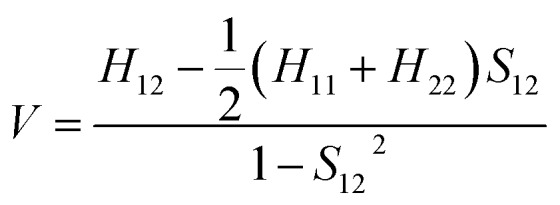

*H* is the self-consistent Hamiltonian matrix of the dimer and *S*_12_ is the overlap integral. The *H* matrix elements are calculated from *H*_*ij*_ = = 〈*φ*_*i*_|*H*|*φ*_*j*_〉, where , where *φ*_*i*_ and *φ*_*j*_ represent the lowest unoccupied molecular orbitals (LUMOs) for electron transport of isolated molecules in the dimer.

## Supplementary Material

Supplementary information

Crystal structure data
