# Supplementary material for: Switching charge-transfer characteristics from p-type to n-type through molecular “doping” (co-crystallization)
Source: Chem Sci. 2016 Feb 25;7(6):3851–6. doi: 10.1039/c5sc04954g (PMC6013807; doi:10.1039/c5sc04954g)
Supplement: Supplementary file 1 [file SC-007-C5SC04954G-s001.pdf]

Electronic Supplementary Material (ESI) for Chemical Science.
This journal is © The Royal Society of Chemistry 2016

Supporting Information

Switching Charge-Transfer Characteristic from *p*-Type to *n*-Type through Molecule "Doping" (Co-crystallization)

Jing Zhang,[†a] Peiyang Gu,[†a] Guankui Long,[a] Rakesh Ganguly,[b] Yongxin Li,[b] Naoki Aratani,[c] Hiroko Yamada,[c] and Qichun Zhang*[a, b]

[a]*School of Materials Science and Engineering, Nanyang Technological University, Singapore*
E-mail: qczhang@ntu.edu.sg
[b]*Division of Chemistry and Biological Chemistry, School of Physical and Mathematical Sciences, Nanyang Techno logical University, Singapore*
[c]*Graduate School of Materials Science, Nara Institute of Science and Technology, Ikoma, Japan*

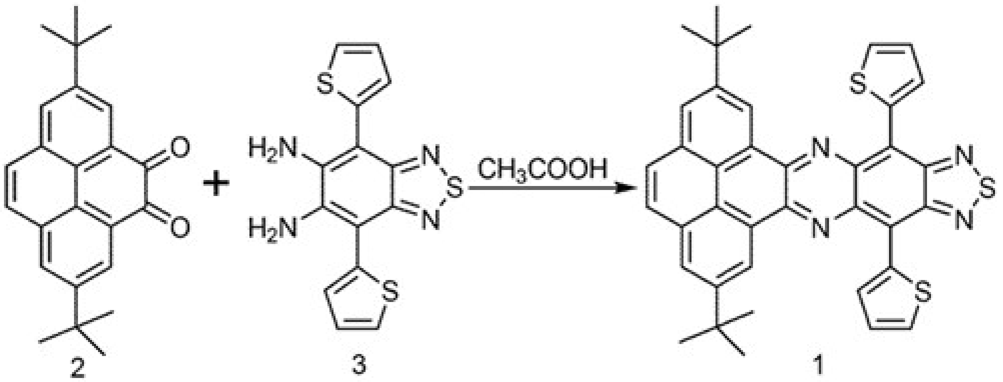

Scheme S1. Synthetic route to DTPTP

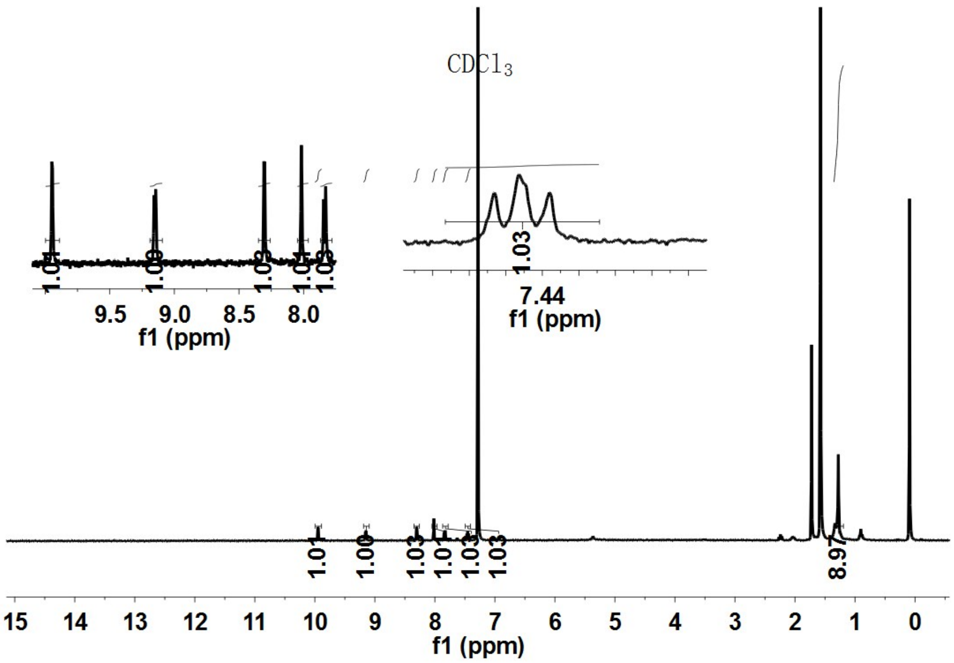

Fig. S1 1H NMR spectrum of DTPTP

^{1}H NMR (300 MHz, $CDCl_3$) δ 9.95 (d, J = 1.8 Hz, 2H), 9.15 (d, J = 3.6 Hz, 2H), 8.30 (d, J = 1.7 Hz, 2H), 8.02 (s, 2H), 7.84 (d, J = 5.2 Hz, 2H), 7.50 – 7.38 (m, 2H), 1.27 (s, 18H).

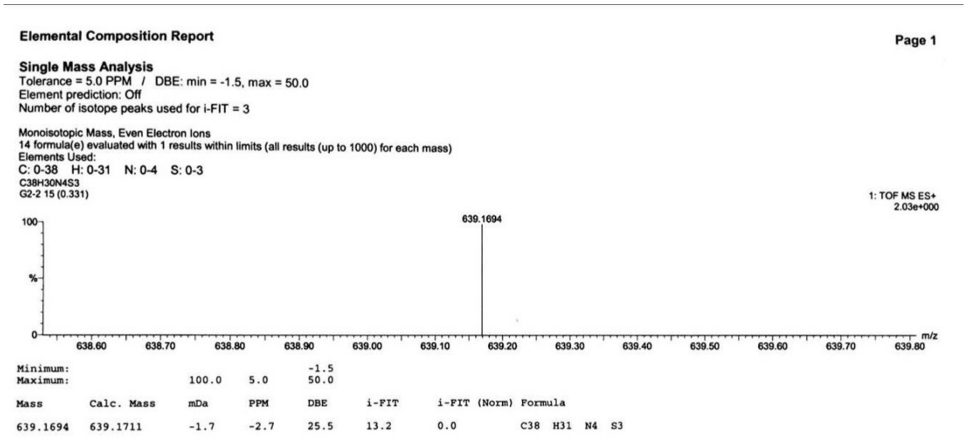

Fig. S2 HR-MS of DTPTP

HR-MS, calcd for $C_{38}H_{31}N_4S_3$, 639.1711; found, 639.1694.

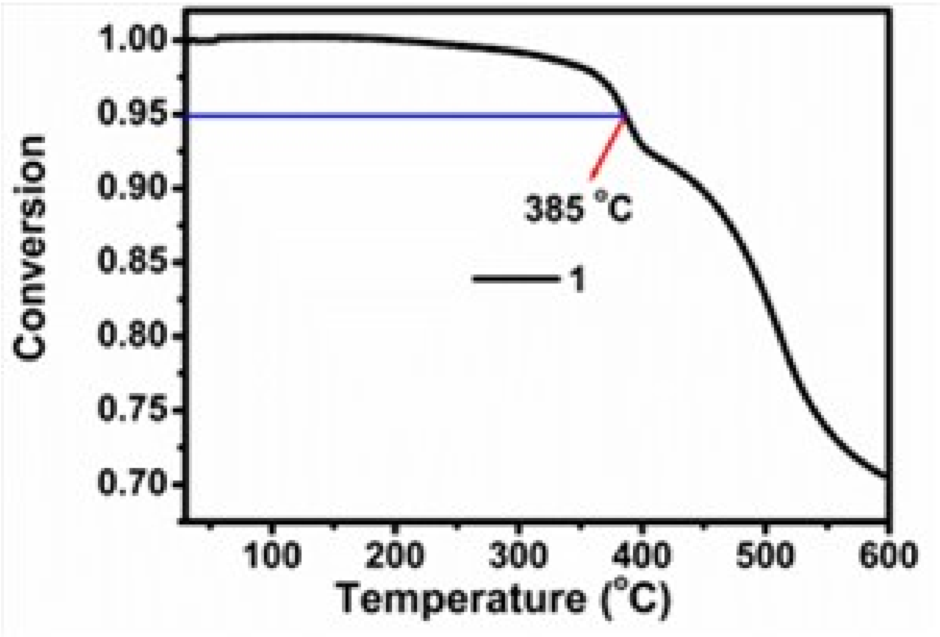

Fig. S3 TGA spectrum of DTPTP

The thermal property of compound DTPTP was evaluated by TGA under nitrogen atmosphere. As we can see, compound DTPTP exhibited very good thermal stability with an onset decomposition temperature of ~ 385 ^{o}C (considering the 5% weight loss temperature).

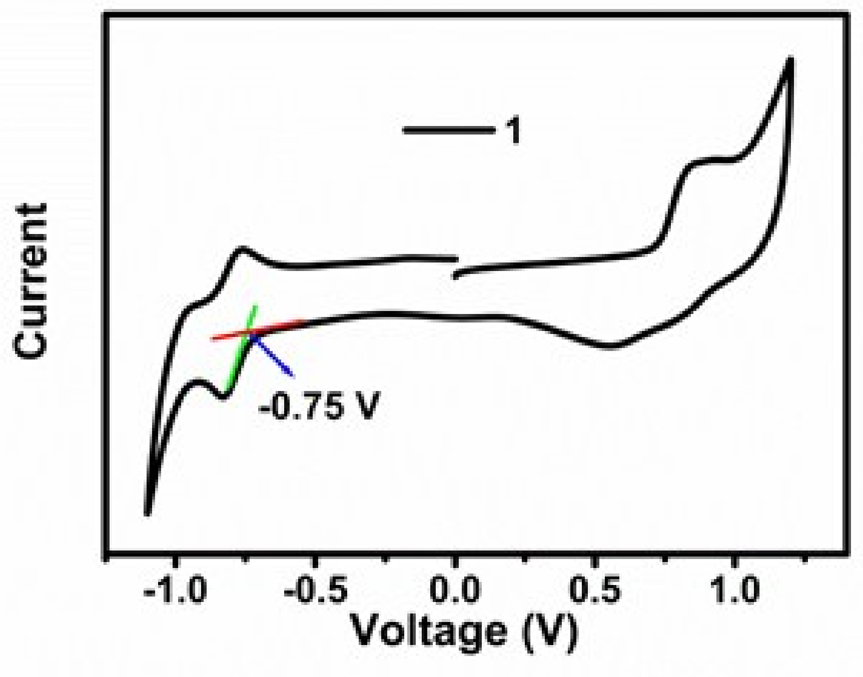

Fig. S4 Cyclic voltammogram curves of compound DTPTP

Cyclic voltammetry was carried out with CHI 604E Electrochemical Analyzer. Glassy carbon (diameter: 1.6 mm; area 0.02 cm^2) was used as working electrode, platinum wires were used as counter electrode and reference electrode, respectively. Potentials were recorded versus Pt in a solution of anhydrous DCM with 0.1 M tetrabutylammonium hexafluorophosphate (n-Bu_4NPF_6) as supporting electrolyte at a scan rate of 100 mV s^{-1}. Fc^+/Fc was used as an internal standard, which has a HOMO energy level of -4.80 eV.

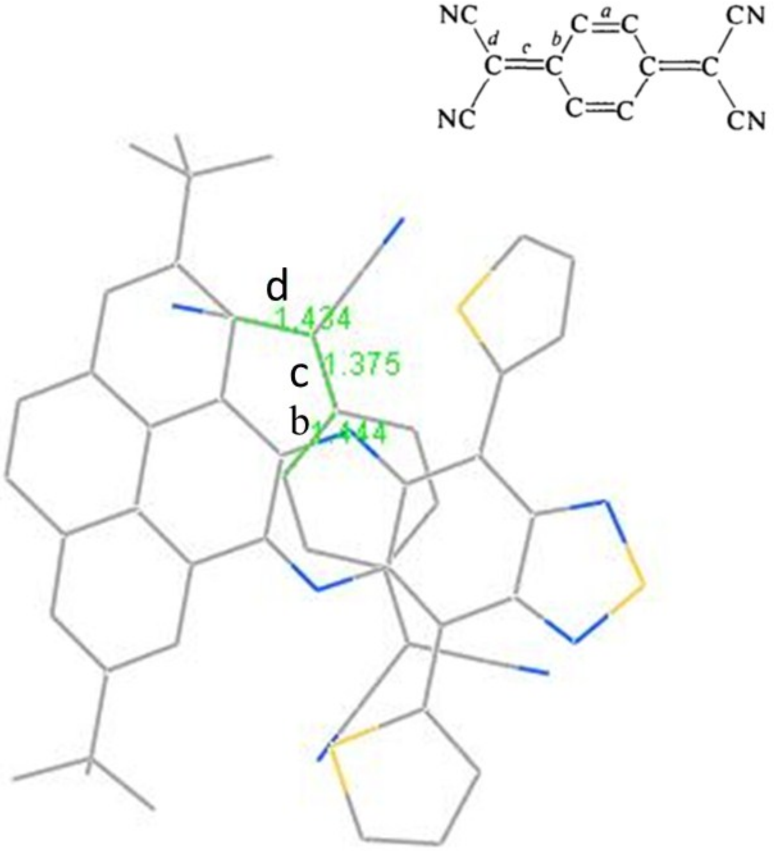

Fig. S5 The bond lengths of TCNQ based on $DTPTP_2$-TCNQ complex indicate that degree of charge transfer in this complex is about 0.1 ($c/(b + d) = 1.375/(1.444+1.434) = 0.478$).

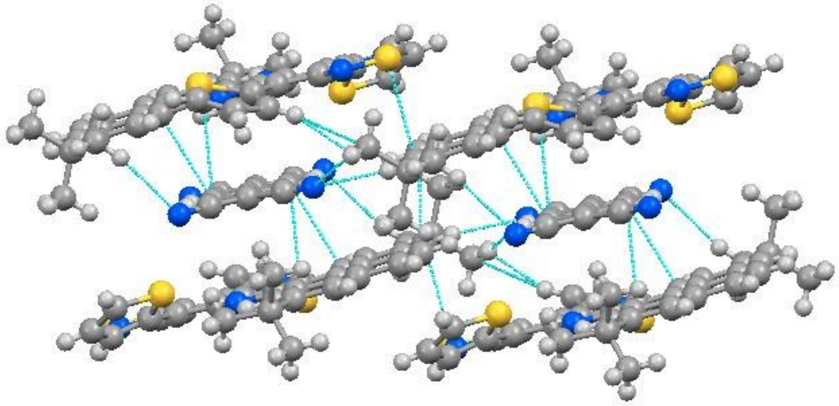

Fig. S6 Short contacts in $DTPTP_2$-TCNQ complex in stacking or neighboring columns

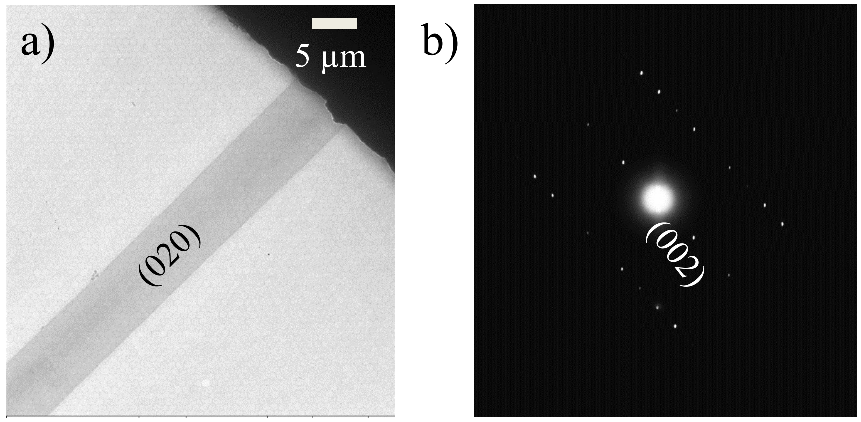

Fig. S7 (a)TEM images and (b) SEAD patterns of DTPTP crystal ribbon. No change of the SAED pattern was observed for the different parts of the same crystal, indicating that the whole ribbon was a single crystal. The SAED pattern of the disk was indexed with the bulk crystal lattice constants.

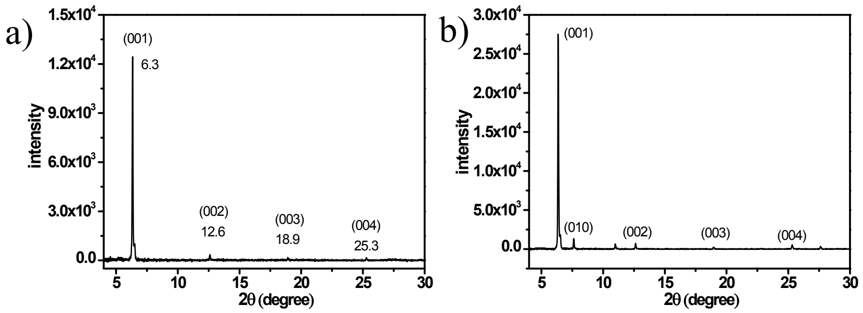

Fig. S8 (a) Perpendicular and (b) tilted X-ray diffraction (XRD) signals from the $DTPTP_2$-TCNQ single-crystalline. We note that the angle of facets at the crystal front end in the optical image (Figure 2c, $\theta \approx 84$ °) corresponds to the angle between the crystal plane (010) and (001) (φ =84.4 °), which implies that the crystal growth orientation is along the stacking direction [100].

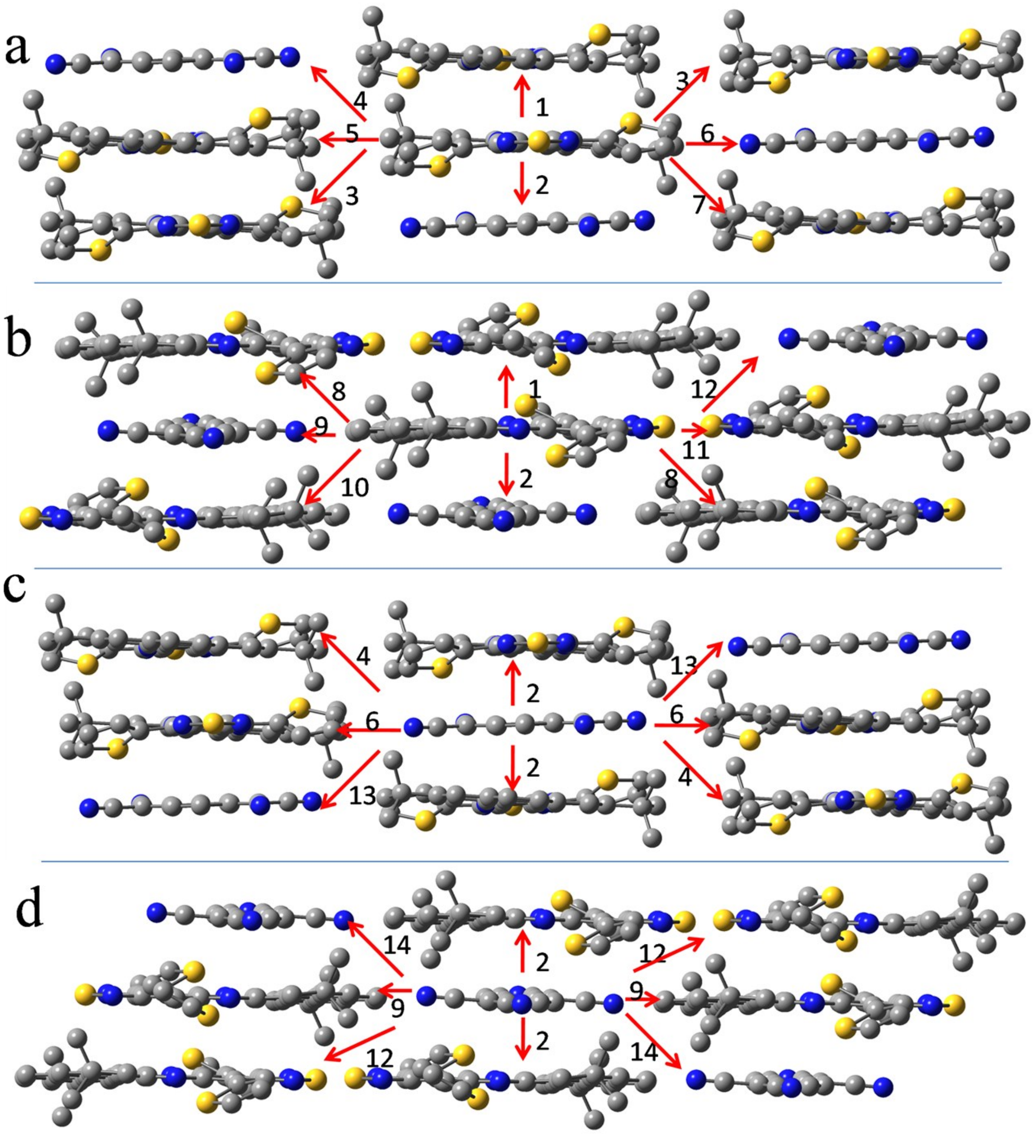

Fig. S9 Hoping routes in the crystal. In (a) and (b), DTPTP is the central molecule, in (c) and (d), TCNQ is the central molecule.

Table S1. The electronic couplings (V) for all the hopping pathways of compound $DTPTP_2$-TCNQ.

pathway	center-center/Å	V_h/meV	V_e/meV
1	4.75	-0.21	-8.36
2	3.22	18.95	-19.58
3	14.48	0.43	0.24
4	14.89	0.00	0.00
5	14.77	1.54	-0.26
6	13.74	-6.11	-3.04
7	13.77	4.33	0.95
8	11.64	2.38	14.74
9	11.54	1.63	2.81
10	12.34	0.00	-0.57
11	14.40	-2.35	25.76
12	15.14	0.00	0.00
13	13.84	0.01	0.01
14	11.64	0.00	0.00

All of the DFT calculations were performed with the Gaussian 09 program package.
